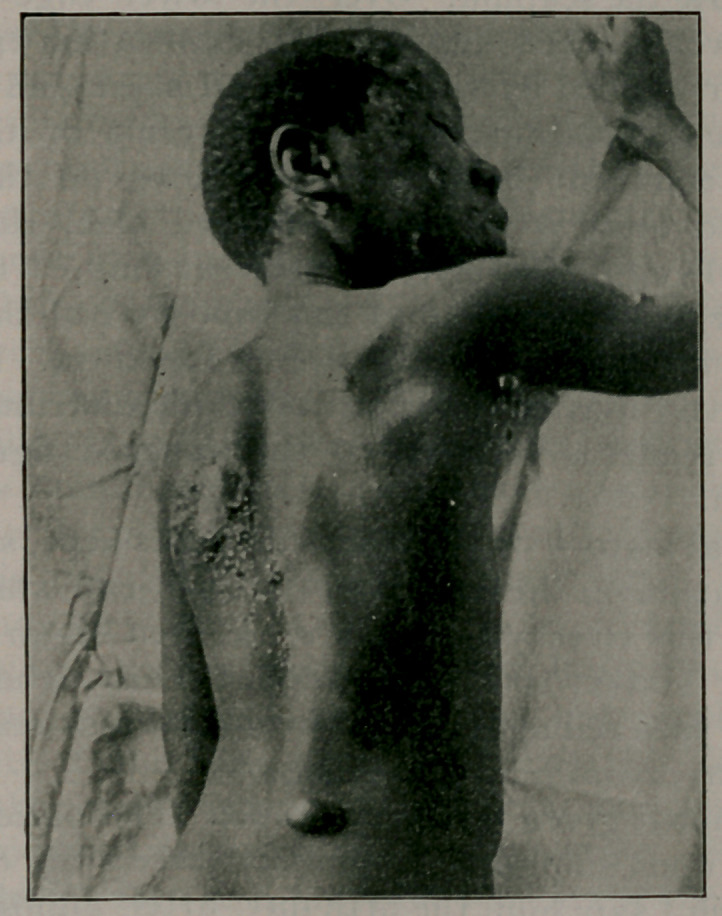# A Case of Pemphigus

**Published:** 1895-10

**Authors:** A. H. Ohmann-Dumesnil

**Affiliations:** St. Louis


### A CASE OF PEMPHIGUS.

BY A. H. OHMANN-DUMESNIL, M. D., ST. LOUIS.

The comparatively small number of cases of this disease observed here, as well as the unusual features occasionally seen, make it a cutaneous affection of more than ordinary interest. In addition to this, the mooted question as to whether it is a contagious affection, due to a bacillus which it has been claimed is associated with it, makes it still more interesting to the student of cutaneous pathology. My intention in reporting a case recently observed by me, is not so much concerned in dealing with the theoretical aspects of the trouble, as it is to deal with the clinical appearances and treatment of the disease. I have purposely chosen an example occurring in the negro for the reason that, no doubt, a number of such occur among the descendants of the African race in the South, where they are much more numerous than north of the Mason and Dixon line. Whilst pemphigus is occasionally seen in Caucasians in this locality, in my experience at least, it has been quite unusual to note it in negroes. Without dealing in any more preliminaries, I will proceed to recount a case which recently came under my observation, and which may be briefly summarized as follows:

Robt. M., colored, an inmate of the House of Refuge, was sent to the St. Louis City Hospital, June 14, 1895. He is a full-blooded negro, thirteen years of age, born in Missouri and having no occupation. Inquiry into his family history developed the fact that both parents are living, as well as four sisters and one brother, and all are healthy. All attempts to discover any hereditary disease were futile. The patient himself declared that he had always been healthy with the exception of an attack of mumps when he was young. He denied the occurrence of all venereal trouble, and does not drink. His hygienic surroundings, of course, had always been poor. Physically, he is a strongly-built lad, and is a good example of a well-developed African. His skin is black, of good grain, and is well distributed over a musculature which betokens marked and well-developed strength.

The history of the present attack is that four weeks previous to his admission to the hospital, he first noticed the eruption, and it kept growing steadily worse up to the time he was seen in the hospital. The bullæ appeared in successive crops and were small at first, gradually increasing in size. At first they were not larger than a large pin's head, but would get the size of a silver dime or quarter dollar in diameter. But little itching was connected with the trouble, although pain on pressure could be perceived. Otherwise the patient felt well, with the exception of a rise in temperature and some headache.

A physical examination showed that all the viscera were normal and properly performing their functions.

I saw the patient on the fifth day of his admission to the hospital, when he presented the following appearance: The integument about the head presented an excoriated appearance, patches freely suppurating being distributed over the face, behind the ears and in the scalp. The eyelids were markedly tumefied and between them exuded a muco-purulent secretion, which was abundant. The anterior part of the neck and upper portion of the chest also presented a number of suppurating excoriations. Lower down on the trunk a number of blebs varying in size from a split pea to a hazel nut presented, and on the abdomen, just below the umbilicus, the largest blebs on the anterior aspect of the body were located. On the arms several blebs also appeared, and in the axillæ the excoriated remains of the former sites of lesions existed, as well as well-defined bullæ. The back was studded with vesicles rather sparsely distributed on the right side, but closely aggregated on the left below the scapula. Over the lower edge of the left scapula a well-marked suppurating excoriation existed, and this was surrounded by vesicles pinhead sized and somewhat larger. The excoriation itself was the result of the bursting of several confluent bullæ. Lower down on the right side there existed quite a large, well-defined bleb, the size of a Guinea fowl's egg, filled with translucent fluid. The contents of the other bullæ observed at this time were of the same nature, but in all they became turbid in a few days. In order to obtain a better idea of the appearance and distribution of the lesions, the reader is referred to the annexed figure from a photograph.

The clinical history of the case, which was kept, showed that the temperature on the entrance of the patient was 102°, remaining at this twenty-four hours. It then began falling, remaining at about 100° until the tenth day, when it rose to 100½°. It then fell again until the normal was reached on the sixteenth day, remaining at this figure.

The treatment given was very simple. The patient was at first placed upon sulphate of quinia in three-grain doses twice daily. When I saw him, the fifth day after admission, I ordered the Asiatic pill made as follows:

℞. Acidi Arseniosi, gr. [illegible].
Pulv Piper Nigris, gr. jss.
Ext. Gentianæ, q s.
M. Ft. pil. no. j.
Sig.:—One such pill after each meal.

In addition to this the bullæ were opened to permit their contents to escape and the following ointment applied to the parts denuded as well as to all the excoriated portions of the skin:

℞. Pulv. Campho-phenique, ʒj.

M. Ung. Aquæ Rosæ, ℥j.

No more crops occurred and the patient made an uninterrupted recovery. He was kept under observation for some time after his recovery before being finally sent back to the House of Refuge. No examinations of the serous contents of the bullæ for micro-organisms were made on account of lack of opportunities to do so.

As will be seen from the above short account, arsenic was relied upon to produce its effects upon the bullar process. So far as my experience goes this remedy acts better upon bullar eruptions than any other one and certainly this would seem to argue against the assumption that pemphigus is a disease due to a specific micro-organism. Those who have found bacteria in pemphigus bullæ do not agree upon the particularity of any one organism, and several have contended that they were saprophytic in character. This latter conclusion is most probably the true one, for, in the case reported, the lesions broke down and their contents escaped. Other inmates of the House of Refuge were exposed to contagion and yet not another case of the disease has been seen at that institution. The conditions prevailing in such an overcrowded as well as not over-cleanly *locale* should certainly favor the transmission of any disease caused by micro-organisms, as a suitable soil for their cultivation is afforded by those very conditions.

While it may be contended that the external application employed is a most excellent germicide, as it has proven itself to be, it must not be forgotten that it was not employed until after the patient had been segregated from his fellows and after a number of the lesions of the disease had broken down and even proceeded to the point of suppuration. So that this could not be invoked as a probable reason for the prevention of a possible contagion.

One point in connection with this case is the rapidity with which recovery established itself. However, it is just in line with my experience with some vesicular, or rather, herpatic troubles concerning which I intend to write at some future time. Treatment in such instances should always be of a more or less radical character, and tentative measures which merely skim the surface should always be avoided, as they only succeed in aggravating the disease, prolonging its duration and making ultimate success more difficult to attain.

## Figures and Tables

**Figure f1:**